# Population and Age-Based Cardiorespiratory Fitness Level Investigation and Automatic Prediction

**DOI:** 10.3389/fcvm.2021.758589

**Published:** 2022-01-05

**Authors:** Liangliang Xiang, Kaili Deng, Qichang Mei, Zixiang Gao, Tao Yang, Alan Wang, Justin Fernandez, Yaodong Gu

**Affiliations:** ^1^Faculty of Sports Science, Ningbo University, Ningbo, China; ^2^Research Academy of Grand Health, Ningbo University, Ningbo, China; ^3^Auckland Bioengineering Institute, The University of Auckland, Auckland, New Zealand; ^4^Medical School, Ningbo University, Ningbo, China; ^5^Faculty of Medicine and Health Sciences, The University of Auckland, Auckland, New Zealand; ^6^Department of Engineering Science, The University of Auckland, Auckland, New Zealand

**Keywords:** physical activity, aerobic capacity, cardiorespiratory fitness, maximal oxygen consumption (VO_2_max), machine learning, support vector machine (SVM)

## Abstract

Maximal oxygen consumption (VO_2_max) reflects aerobic capacity and is crucial for assessing cardiorespiratory fitness and physical activity level. The purpose of this study was to classify and predict the population-based cardiorespiratory fitness based on anthropometric parameters, workload, and steady-state heart rate (HR) of the submaximal exercise test. Five hundred and seventeen participants were recruited into this study. This study initially classified aerobic capacity followed by VO_2_max predicted using an ordinary least squares regression model with measured VO_2_max from a submaximal cycle test as ground truth. Furthermore, we predicted VO_2_max in the age ranges 21–40 and above 40. For the support vector classification model, the test accuracy was 75%. The ordinary least squares regression model showed the coefficient of determination (*R*^2^) between measured and predicted VO_2_max was 0.83, mean absolute error (MAE) and root mean square error (RMSE) were 3.12 and 4.24 ml/kg/min, respectively. *R*^2^ in the age 21–40 and above 40 groups were 0.85 and 0.75, respectively. In conclusion, this study provides a practical protocol for estimating cardiorespiratory fitness of an individual in large populations. An applicable submaximal test for population-based cohorts could evaluate physical activity levels and provide exercise recommendations.

## Introduction

The WHO identified physical inactivity as the fourth leading risk factor for non-communicable diseases accounting for high-mortality rates every year (1). Physical inactivity may cause heart disease, stroke, colon cancer, breast cancer, depression, and anxiety (2). Furthermore, low cardiorespiratory fitness has been associated with all causes of mortality and is an independent predictor of cardiovascular diseases (3).

The WHO recommends adults aged 18–64 years should attend regular moderate-intensity (≥150–300 min) or vigorous-intensity (≥75–150 min) aerobic physical activity per week (4). According to the Lancet Physical Activity Series Working Group (5), around 30% of adults worldwide are physically inactive, and inactivity rises with age. Males and females also occupy different proportions, and physical inactivity appears more in women than men (5). Apart from a range of chronic diseases and early deaths associated with the pandemic of physical inactivity, it also causes a substantial economic burden (6, 7). According to a study by Ding et al. (8), physical inactivity cost more than 50 billion dollars in the healthcare system in 2013 globally. Evaluating cardiorespiratory fitness levels is crucial in preventing physical inactivity, and it can be achieved by measuring or predicting the maximal oxygen consumption (VO_2_max).

Aerobic capacity is a quantifiable indicator of physical fitness level (9). VO_2_max reflects the aerobic capacity and maximal cardiorespiratory function, and it is critical for assessing cardiorespiratory fitness and physical activity level. Matsuo et al. (10) found that physical activity levels of the workers (age: 30–60 years) are significantly correlated with VO_2_max. People (age: 48.5 ± 14.4 years) meeting the physical activity criteria of 150 min/week of the daily moderate-intensity exercise demonstrated approximately 10% higher VO_2_max than their counterparts (11). Better cardiorespiratory fitness is associated with a lower risk of all-cause mortality (12, 13). A 34-year follow-up study found life-long physical activity may reduce the risk of breast cancer among female teachers aged ≥ 25 years (14). Low levels of physical activity and low cardiorespiratory fitness are the indices of the development of metabolic syndrome in both male and female adolescence (15).

It is crucial to estimate VO_2_max utilizing a simple, valid, and reliable method for epidemiologic studies of physical activity (16). VO_2_max can be measured by direct gas analysis and submaximal cycle ergometry. Direct measurement of VO_2_max from gas analysis using a progressive exercise test demands a maximal effort from the subject restricted to a well-equipped laboratory environment and technical expertise (17). Hence, maximal exercise tests on a treadmill or cycle ergometer are unappealing to some individuals, and some elderly or physically inactive individuals may endure a high risk to undertake the test. Astrand-Rhyming is one of the most administered submaximal cycle ergometry tests. It calculates VO_2_max by evaluating the steady-state heart rate (HR) during a 6-min submaximal test. The test protocol and results also exhibited good validity and reliability in healthy populations (17, 18). Hoehn et al. (18) showed that the difference between the estimated VO_2_max from the Astrand-Rhyming cycle ergometer test and VO_2_max from the maximal cycle ergometer test was <1 ml/kg/min. Test-retest reliability analysis over 1 week also showed no mean difference (17). Huerta et al. (9) evaluated physical fitness among Israeli soldiers using the Astrand-Rhyming 6-min cycle ergometer test and found a sex-specific difference of the estimated VO_2_max.

Machine learning is the study of computer algorithms that improve automatically through experience and by the use of data (19). Machine learning has been widely utilized in health informatics and physical fitness investigation in the recent years (20, 21). Machine and deep learning techniques have accelerated human experiments and tests conducted from the laboratory to the real world in the past decades (22–25). Inertial wearable sensors combined with machine learning algorithms could predict and evaluate biomechanics performances and energy expenditure (26–28). Human activity, such as walking, running, sitting, and cycling, could be accurately detected and classified using supervised learning methods (29–31). Therefore, sedentary behavior and physical activity level can be estimated by wearable technology (27). AI-Mallah et al. (32) demonstrated that cardiorespiratory fitness data could predict all-cause mortality by classifying the data into predetermined categories using the K-Nearest Neighbor (KNN) algorithm. Sakr et al. (33) found that the cardiorespiratory fitness data could be used to predict the prevalence rate of hypertension.

Predicting VO_2_max by machine learning approaches is emerging recently. Anthropometric parameters, time of exercise, workload, and HR self-reported rating of perceived exertion (RPE) are commonly used variables or predictors (34–36). Beltrame et al. (35) revealed that machine learning algorithms successfully predicted VO_2_max of forty-five health participants during the early stages of the test at maximal cardiopulmonary exercise testing. Machine learning methods can also predict VO_2_max based on a 20 m shuttle run test with root mean square error (RMSE) <5.5 ml/kg/min with a sample size of 308 (37). Artificial neural networks (ANNs) could predict VO_2_max based on a single-stage submaximal exercise test of 126 healthy adults (34). Support Vector Machine (SVM) estimated VO_2_max of 100 healthy participants also achieved high accuracy (36, 38).

However, the previous studies were conducted from relatively small sample sizes and predicted VO_2_max based on general healthy participants. Furthermore, no study validates the prediction accuracy of cardiorespiratory fitness between age-based populations. Therefore, this study aimed to classify and predict the population-based (university teachers) cardiorespiratory fitness based on anthropometric parameters, workload, and steady-state HR of the submaximal exercise test. It was hypothesized that: (1) VO_2_max is correlated with physical fitness levels and could be predicted and classified by physical fitness tests; (2) increased age contributed to the decreased cardiorespiratory fitness in age-based groups and it can be predicted.

## Materials and Methods

### Participants

This study evaluated cardiorespiratory fitness among a large population and aged-based sample. Five hundred and seventeen (255 males and 262 females, age: 40.75 ± 9.16 years; height: 165.40 ± 7.70 cm; weight: 63.49 ± 11.33 kg; BMI: 23.09 ± 3.04 kg/m^2^) university teachers from a university in the southeast of China were recruited into our study. Anthropometric parameters contain height, weight, and BMI. Maximal HR was calculated based on the age-predicted maximal HR: max HR = 220–age. All participants were healthy and free of any medical condition that may potentially affect VO_2_max and exercise activities. All subjects were informed of the purpose, requirements, and details of this study, and written consent was obtained from each participant before the test. The study was approved by the ethics committee in Ningbo University (RAGH20190825).

### Experimental Setting and Test Protocol

All cardiopulmonary aerobic tests were conducted from April 2020 to July 2020. Monark ergometer (928E, Varberg, Sweden) was utilized for the Astrand-Rhyming 6 min cycle ergometer test. The approach estimates aerobic capacity based on HR and power during the sub-max intensity test on the cycle ergometer. Each participant was given 5 min to warm up and familiarize the test environment. Participants were introduced to adjust the heights of the cycle ergometer seat and handlebar before the test. Borg's rating of perceived exertion (RPE) scale was adopted to monitor fatigue. The speed of the cycle ergometer was set at 50 r/min before the test. The whole test lasted 6 min, and during the first 3 mins, the workload was adjustable to obtain the stable HR between 125 and 175 beats/min (approximately 75% max HR). HR was monitored during the tests by a Polar Electro (H10, Kempele, Finland). Test workload was recorded and accepted HR was evaluated for each participant. VO_2_max was estimated using the Astrand-Rhyming nomogram based on cardiac response to 6 min of constant submaximal cycle work (9). Each participant's cardiorespiratory fitness was further divided into poor, average, good, and excellent classes based on measured VO_2_max in Monark 1.0.15.0 (Vansbro, Sweden).

### Machine Learning Approaches

This study initially classified the aerobic capacity followed by VO_2_max predicted using an ordinary least squares regression model with measured VO_2_max from submaximal cycle test as ground-truth. Furthermore, we predicted VO_2_max in the age 21–40 years and above 40 years groups. All predictors were z score normalized with a mean value of 0 and a standard deviation of 1 (39). XGBoost, KNN, logistic regression, decision tree, random forest, Naïve Bayes, and SVM algorithm were considered for this classification task. KNN assigned ten neighbors and used the standard Euclidean metric. The logistic regression algorithm employed the penalty algorithm of L1 and the LibLinear algorithm as the solver. We chose the kernel and C in the SVM as linear and 10, respectively. Gaussian naïve Bayes was picked in this study. The entropy criterion was selected for the decision tree and random forest algorithms, with maximum tree depth and the number of trees as 10 and 100, respectively. The number of trees, maximum tree depth, and learning rate were set as 10, 100, and 0.1, respectively, in the XGBoost model. SVM with linear kernel was selected as it performed the best prediction accuracy of the 10-fold cross-validation model. Based on the performance of the cycle ergometer test, cardiorespiratory fitness was classified into the following four categories: poor, average, good, and excellent.

Data were split into 60, 20, and 20% for training, validation, and testing, respectively. Twenty percent of the raw dataset was initially selected for testing. The remained 80% of the data was used for training and validation. Then, we further divided training and validation into the ratio of 75 and 25%. The SVM algorithm constructs hyperplanes between categories by maximizing the margin using support vectors. The soft margin parameter of C balances the trade-off between margin width and misclassification rate (40). To achieve a good trade-off between training and test accuracies and avoid underfitting and overfitting problems, we performed hyperparameter tuning based on four-fold grid search cross-validation (GridSearchCV) on the training dataset to determine the best C parameter of support vector classification (SVC) model from range 0.01, 0.1, 1, 10–100 [37]. Finally, C = 10 was selected as the best parameter and adopted for the test dataset.

The ordinary least squares regression was employed to predict VO_2_max of each participant. For classification and regression, gender, age, height, weight, body mass index (BMI), maximal HR, test workload, and accepted HR were adopted as predictors. The SVM classification model was evaluated by accuracy, precision, recall, F1-score, and Matthews correlation coefficient. The linear regression model was estimated by Pearson product-moment correlation coefficients (R^2^), the mean absolute error (MAE), and RMSE. The coefficient of each predictor was extracted to rank the feature importance of the regression model.

### Statistical Analysis

Participants were divided into four groups, age 21–30, 31–40, 41–50, and above 50. The accepted HR, test workload, and aerobic capacity between groups were compared utilizing ANOVA analysis in R (version 4.0.5, R Foundation for Statistical Computing, Vienna, Austria). Tukey's honest significance differences (HSD) *post hoc* analysis was used to conduct statistical analysis of VO_2_max between groups with the significance level *p* < 0.01.

## Results

### The Anthropometric and Submaximal Test Information

The anthropometric and cycle ergometer test information is presented in Table 1. The accepted HR during tests was decreased with the increase of age (from 137.6 ± 11.6 bpm in the age 21–30 group decreased to 133.8 ± 11.5 bmp in the age > 50 group), but no statistical significance (*F* = 2.6, *p* > 0.05). The test workload was gradually decreased with increased age in 21–30, 31–40, and 41–50 age groups (646.6 ± 198.8, 609.8 ± 190.3, and 568.4 ± 172.8 kpm/min, respectively). Also, there was no significant difference presented (*F* = 1.7, *p* > 0.05).

**Table 1:** The anthropometric and cardiopulmonary aerobic test information in different age groups (data was shown in mean ± SD).

	**Age (years)**	**Height (cm)**	**Weight (kg)**	**BMI (kg/cm^**2**^)**	**Test workload (kpm/min)**	**Accepted HR (bpm)**	**VO_**2**_max (ml/kg/min)**
21–30 (*n* = 29)	27.5 ± 1.5	168.6 ± 7.4	63.9 ± 11.1	22.4 ± 2.7	646.6 ± 198.8	137.6 ± 11.6	46.7 ± 11.5
31–40 (*n* = 192)	35.0 ± 2.7	166.4 ± 8.1	63.1 ± 12.3	22.6 ± 3.2	609.8 ± 190.3	136.2 ± 10.9	43.5 ± 11.3
41–50 (*n* = 159)	44.6 ± 2.9	164.6 ± 7.0	62.9 ± 10.7	23.1 ± 3.0	568.4 ± 172.8	135.8 ± 11.0	38.8 ± 9.3
>50 (*n* = 137)	54.9 ± 3.2	164.3 ± 7.7	64.6 ± 10.7	23.8 ± 2.8	583.6 ± 155.9	133.8 ± 11.5	36.8 ± 9.2

*HR, heart rate; VO_2_max, maximal oxygen consumption*.

### VO_2_max in the Different Age Groups

**Figure 1:**
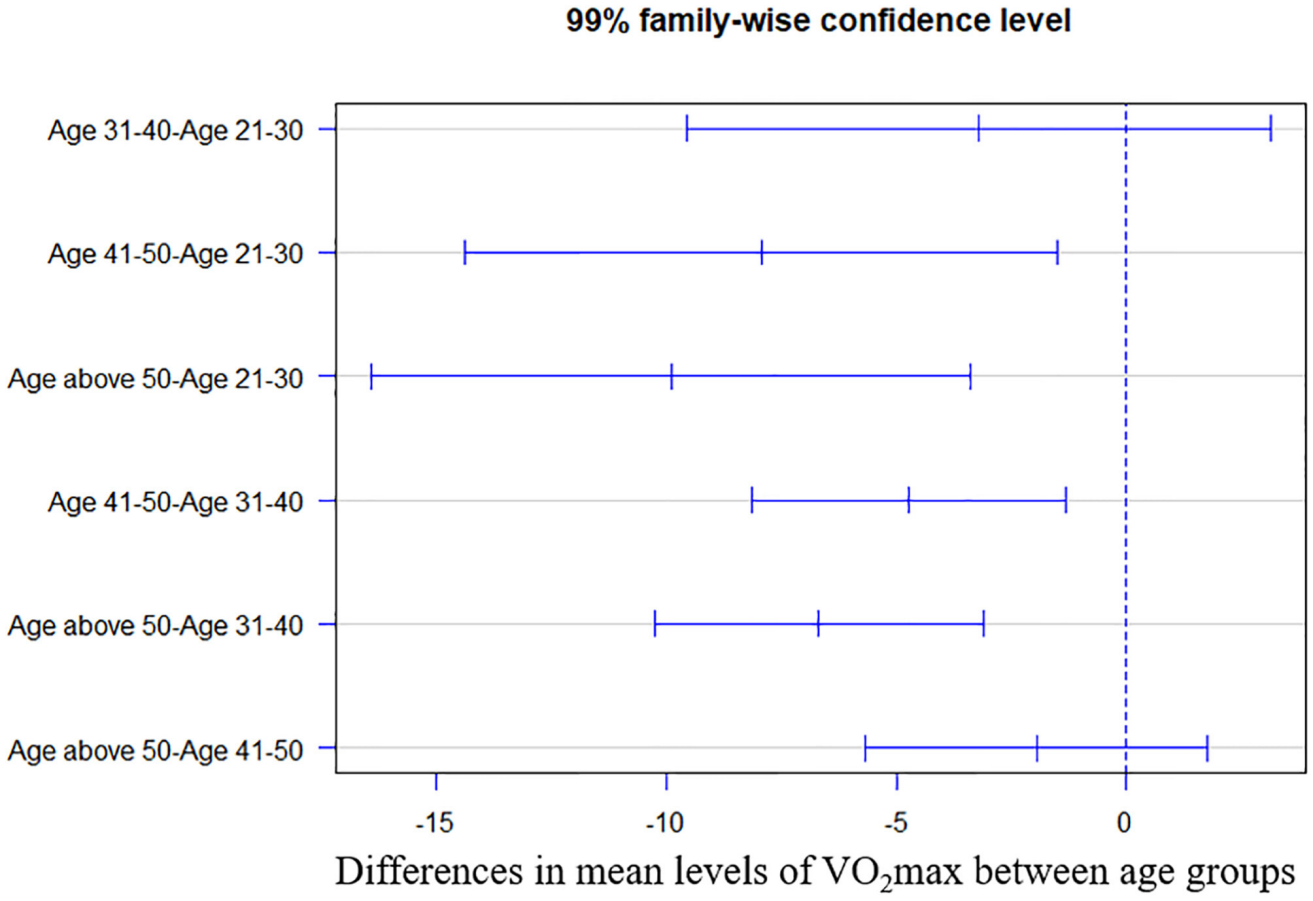
Tukey's honest significance differences test of maximal oxygen consumption (VO_2_max) between age groups.

The ANOVA analysis of VO_2_max shows the statistical difference with *F* = 16.6 and *p* < 0.01. Tukey's HSD *post-hoc* analysis with 99% CI was demonstrated in Figure 1. Compared with the age 21–30 group, VO_2_max in the age 41–50 and >50 groups were significantly decreased (both *p* < 0.01, mean difference: −7.9 and −9.9, 99% CI: −14.4 to −1.5 and −16.4 to −3.4). VO_2_max was also significantly different in the age 41–50 (*p* < 0.01, mean difference −4.7, 99% CI: −8.1 to −1.3) and above 50 groups (*p* < 0.01, mean difference −6.7, 99% CI: −10.2 to −3.1) when comparing to the age 31–40 group.

### The Performance of the Classifier

**Figure 2:**
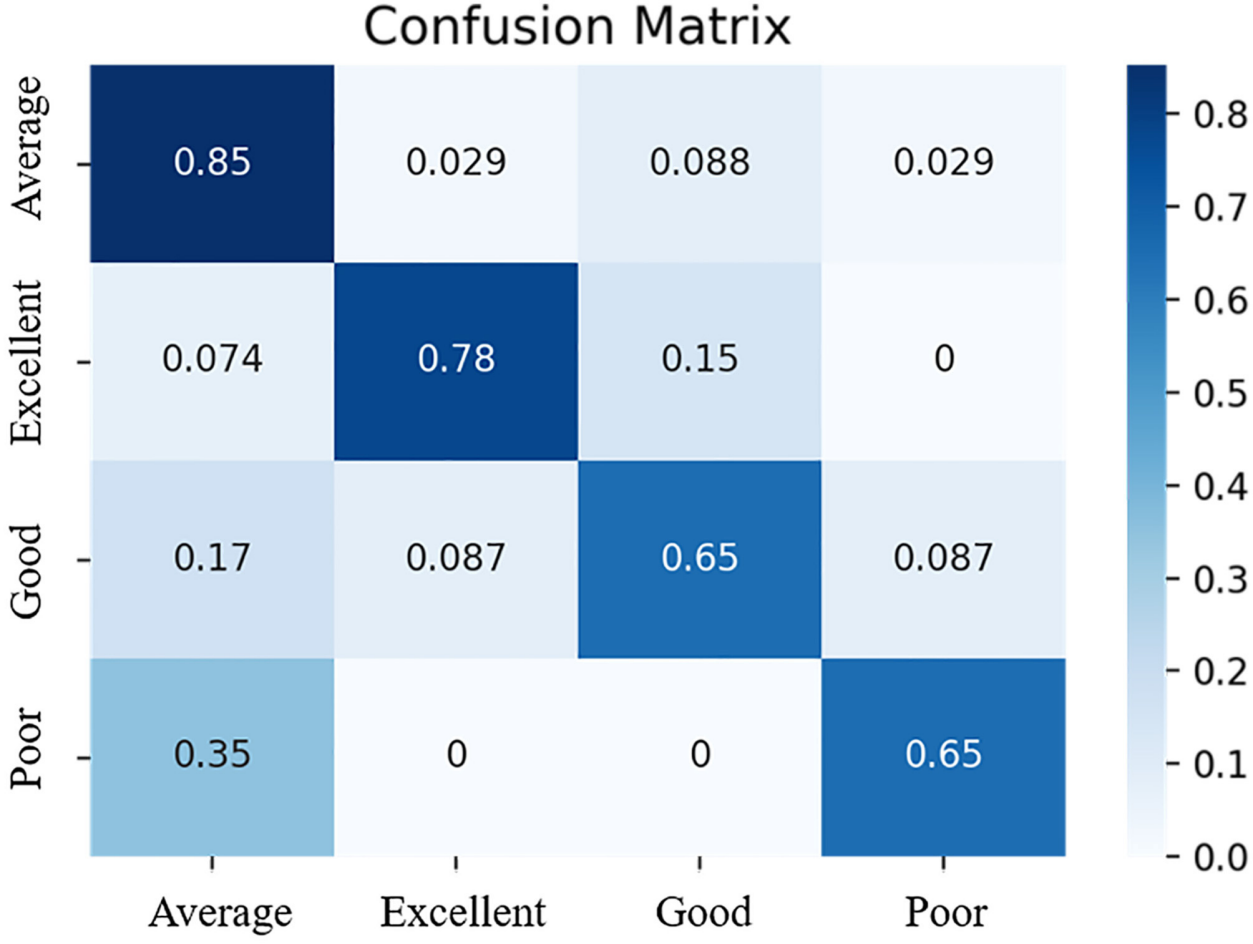
Confusion matrix of the SVM classification model based on VO_2_max differences.

The four-fold cross-validation accuracy was 76%, and the accuracy in the test dataset was 75%. The precision, recall, F1-score, and Matthews correlation coefficient are presented in Table 2. The accuracies of VO_2_max of the classifying participants into poor, average, good, and excellent categories were shown in the confusion matrix (Figure 2). The X-axis showed the actual level of VO_2_max, while the Y-axis depicted the predicted VO_2_max utilizing an SVM classifier, and values were normalized to present the percentage of each class. The average level of VO_2_max demonstrated the highest accuracy followed by the excellent level with 85 and 78%. The accuracies in the good and poor performances were relatively lower with 65 and 65%.

**Table 2:** The classification report of linear SVC classifier.

	**Physical performance**	**Number of observations**	**Cross-Validation accuracy**	**Accuracy**	**Precision**	**Recall**	**F1-Score**	**Matthews correlation coefficient**
Validation dataset	Poor	59	0.76		0.74	0.73	0.74	
	Average	121			0.72	0.74	0.73	0.66
	Good	117			0.70	0.67	0.68	
	Excellent	116			0.83	0.87	0.85	
Test dataset	Poor	20		0.75	0.81	0.65	0.72	0.66
	Average	34			0.69	0.85	0.76	
	Good	23			0.68	0.65	0.67	
	Excellent	27			0.88	0.78	0.82	

### The Linear Regression Model

**Figure 3:**
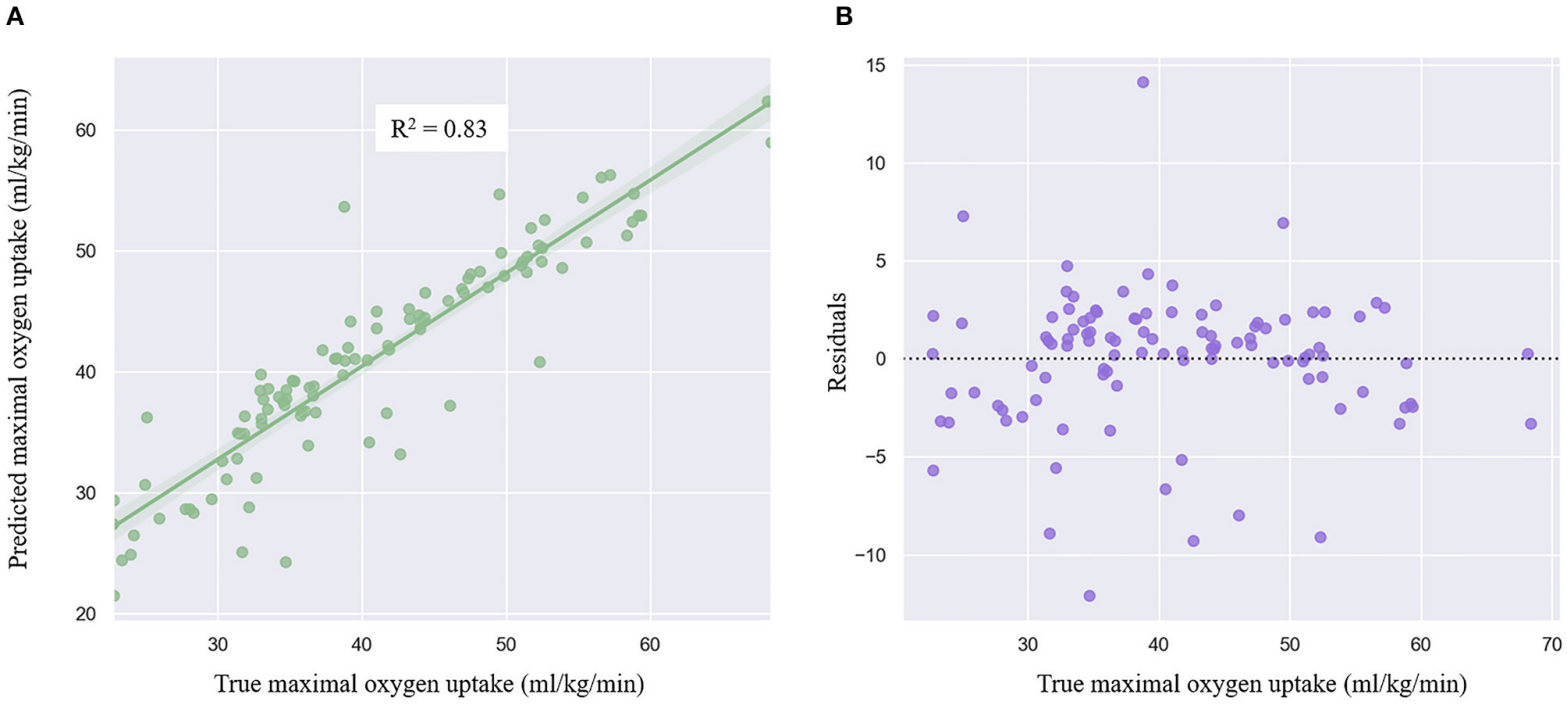
Regression plot **(A)** and residuals plot **(B)** of the linear regression model.

**Figure 4:**
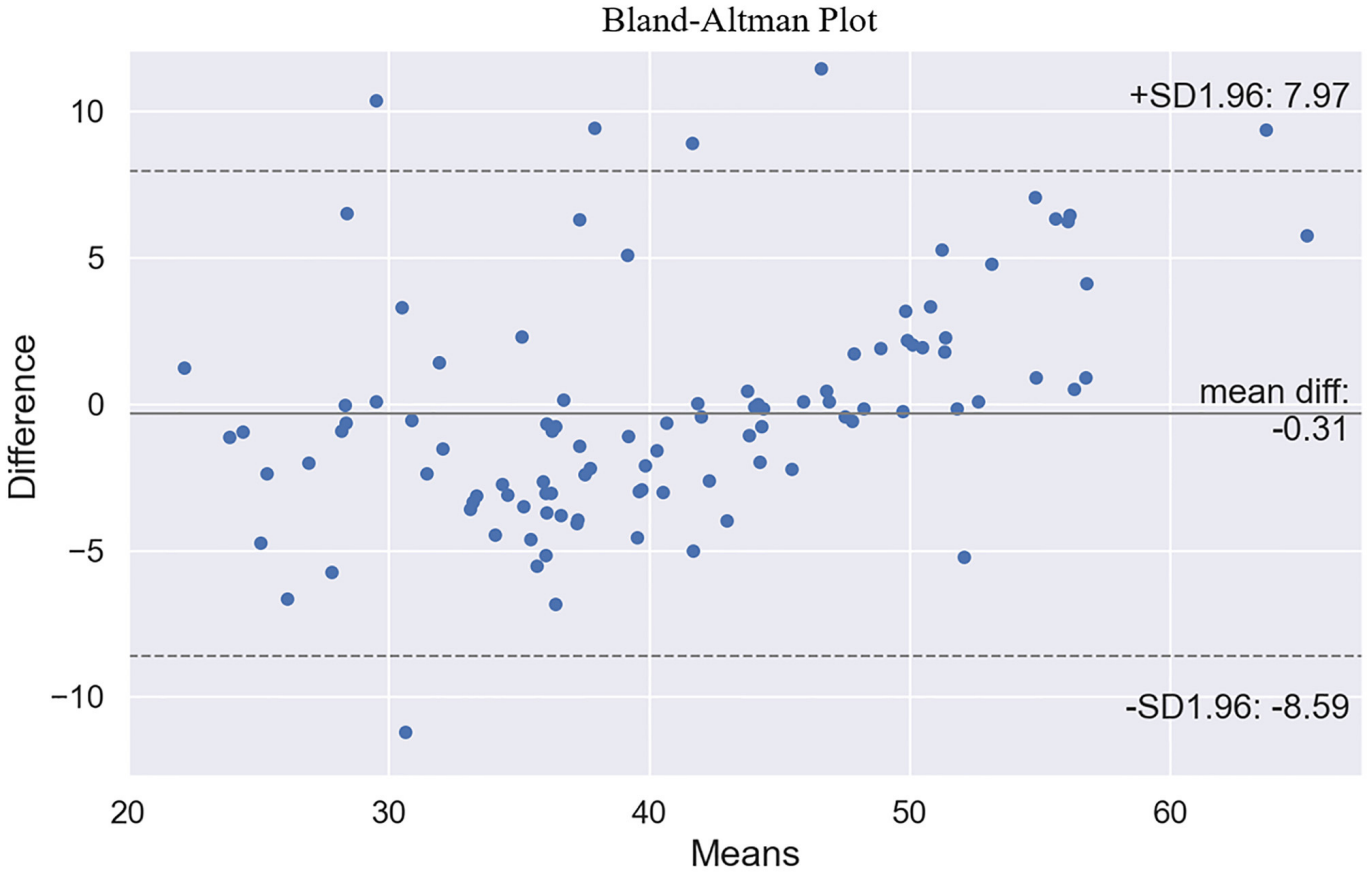
Bland-Altman plot of true and predicted maximal oxygen uptake.

Gender, BMI, and height were the three most crucial input variables that contribute to performance of the model, followed by accepted HR, weight, age, maximal HR, and test workload. The ordinary least squares regression model showed the coefficient of determination (*R*^2^) between actual and predicted VO_2_max in the validation dataset was 0.81 and 0.83 in the test dataset (Figure 3A). MAE and RMSE were 3.37 and 4.45 ml/kg/min in the validation dataset, and 3.12 and 4.24 ml/kg/min in the test dataset. Figure 3B demonstrated the residuals plot of predicted VO_2_max compared with the true VO_2_max. The Bland-Altman plot exhibited the mean difference and 95% limits of agreement between the observed and predicted VO_2_max (Figure 4). The mean difference was very close to 0, which is −0.31, and most of the points are scattered in the ±1.96 SD (above −8.59 ml/kg/min and below 7.97 mm/kg/min).

**Figure 5:**
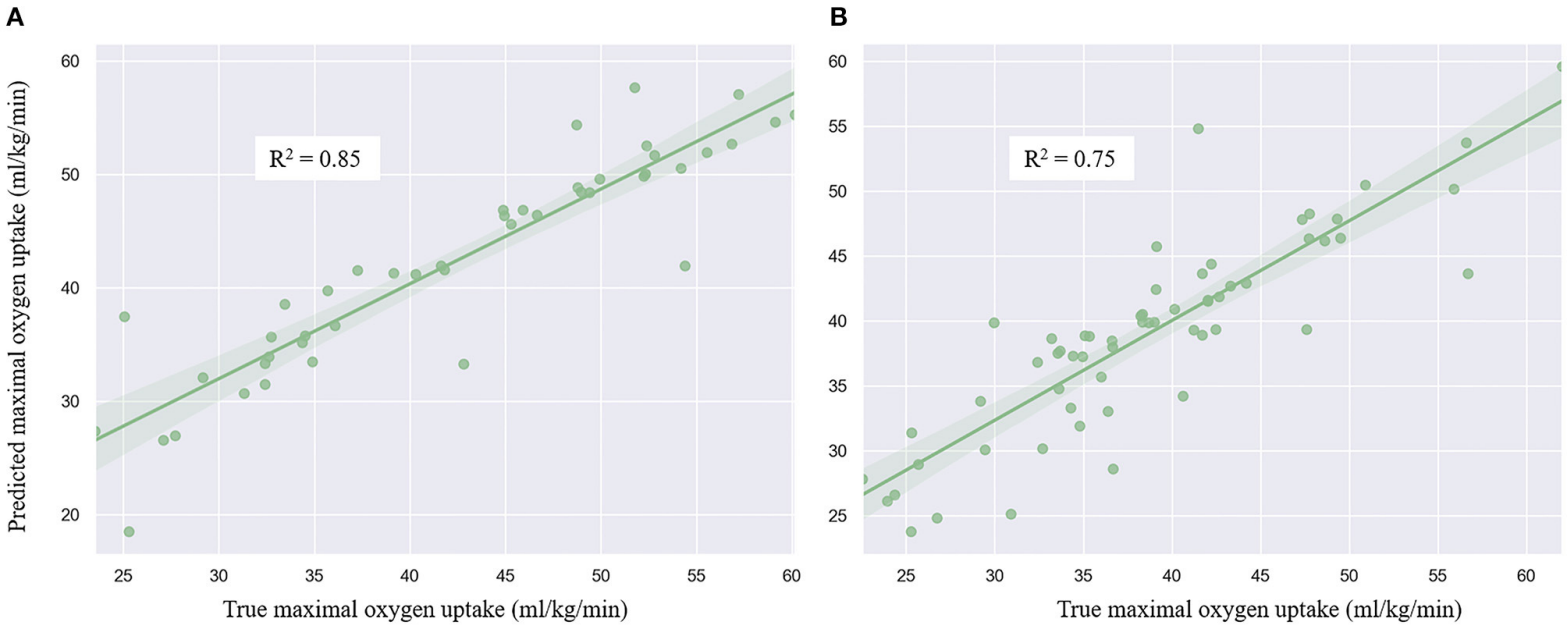
Regression plot of the linear regression model of age 21–40 group **(A)** and age above 40 group **(B)**.

The coefficient of determination between the true and predicted VO_2_max in the validation and test datasets (Figure 5A) of the age 21–40 group was *R*^2^ = 0.8 and 0.85, respectively. MAE and RMSE in the test dataset were 2.72 and 4.02 ml/kg/min, respectively. Figure 5B presented *R*^2^ = 0.75 of the ordinary least squares regression model in the age ≥ 40 group of the test dataset, with MAE = 3.2 ml/kg/min and RMSE = 4.26 ml/kg/min in the test dataset.

## Discussion

VO_2_max is an essential part of health and physical fitness. This study predicted the VO_2_max based on anthropometric parameters and the cycle ergometer test utilizing machine learning. Furthermore, we estimated that the ordinary least squares regression model in younger and older groups. It was found the SVC algorithm could successfully classify the cardiorespiratory fitness level into four classes (i.e., poor, average, good, and excellent). The linear regression model can predict VO_2_max with gender, age, height, weight, BMI, maximal HR, test workload, and accepted HR as predictors. The ordinary least squares regression performance is better in the younger group (21–40 years old, *R*^2^ = 0.85) than the group with age above 40 years (*R*^2^ = 0.75).

Previous studies assessed and predicted VO_2_max using different predictors and test protocols (34–38), but no study classifies the aerobic capacity into subcategories in the previous work. SVM exhibited outstanding performance in the classification tasks (36, 38, 40, 41). This study adopted it as the classifier to separate the aerobic capacity. According to the findings of this study, the VO_2_max level is predictive. It can be classified based on the anthropometric parameters and workload and steady-state HR of the submaximal exercise test.

This study identified the anthropometric measurements that can be directly employed to evaluate cardiorespiratory fitness. The accuracy would be further improved by incorporating exercise parameters of an individual (i.e., HR during exercise and workload). Therefore, it provides a practical protocol for estimating cardiorespiratory fitness of an individual in large populations. By monitoring VO_2_max in the different age groups, people will better understand their health condition and improve cardiovascular endurance. On the other hand, people can be guided specifically to promote health.

Our aging population presents a significant health challenge globally (42–44). The medical and healthcare system is under increased pressure with an increased economic burden (8). Estimating and predicting physical activity may play a crucial role nowadays in lessening the burden on the healthcare system. Monitoring and evaluating physical activity or cardiorespiratory fitness levels among populations, especially for the elderly, would help guide policies to increase activity levels of the populations and reduce the burden of non-communicable diseases in the public health system (5).

Przednowek et al. (37) predicted VO_2_max based on a 20 m shuttle run test using ordinary least squares regression, ridge regression, Lasso regression, and ANNs. The results showed that the models for females generated less error than males, but only young participants (mean age: 20.6) were included in the study. Akay et al. (34) estimated VO_2_max of 126 participants utilizing ANNs (*R*^2^ = 0.94) with anthropometric parameters, steady-state HR of jogging, and jogging speed as predictors. The age range of the subjects, however, was from 17 to 40 years. VO_2_max of Children and adolescents can be predicted in a submaximal run test using multiple linear regression and ANNs models (45). Huerta et al. (9) employed the cycle ergometry evaluating VO_2_max among a large population-based sample of Israeli men and women with age ranging from 18 to 25 years. The submaximal cycle ergometry test has been developed as a valuable tool to estimate cardiorespiratory fitness and VO_2_max due to its lower cost, lower test risk of complications, and being applicable for the elderly (34). However, previous studies only estimated or predicted physical fitness of young adults. The ordinary least squares regression model in this study showed that VO_2_max is also predictable for the older population and predicted decreased cardiorespiratory fitness level with increased age, which is consistent with the previous study that physical inactivity raises with age (5).

It also showed that the predicted performance of the cycle ergometer test method is affected by age. Due to physiological changes, the elderly might potentially take less physical exercise or physical activity, and aerobic capacity is decreased with age (5). The cycle ergometer test exhibited relatively poor ability to predict VO_2_max. Using this approach to predict the VO_2_max in the younger population may be better than in the older age group. It is recommended to explore the replaceable method to estimate and predict the VO_2_max among the elderly population in the future. This study also found the cardiovascular endurance is significantly decreased for people aged more than 40 years. Physical inactivity in this period may contribute to decreased VO_2_max. Physical activity recommendations or exercise protocol should consider providing specific guidelines for this age group in the future.

Some limitations should be noted, despite the promising findings in this study. Although we predicted the cardiorespiratory fitness between the younger and older populations, the sample size of cohorts in the age 21–30 and age above 60 groups is relatively small. Thereby, future studies should add more elderly participants to investigate their physical activity and cardiorespiratory fitness level. As physiological mechanisms differ between males and females, sex difference should also be taken into consideration. On the other hand, due to directly measuring individuals age above 40 years has the higher medical risk, VO_2_max was measured from a submaximal test rather than maximal exertion test in this study. Furthermore, the ordinary least squares regression model was employed in this study. Other non-linear regression models may improve the performance of the model and decrease errors. Future studies should also attempt to predict VO_2_max from larger sample sizes or consider including synthetic data.

## Conclusion

In conclusion, this study investigated population and age-based cardiorespiratory fitness levels and classified and predicted VO_2_max using classification and linear regression approaches. The findings showed that VO_2_max level is predictive and can be classified based on the anthropometric parameters and workload and steady-state HR of a submaximal exercise test. Physical activity or exercise recommendations could be given to the university teachers by conducting an applicable submaximal test. Although the regression model exhibited reasonable accuracy in predicting the aerobic capacity among population above age 40 years, it is worth exploring a more comprehensive model or test protocol to estimate the cardiorespiratory fitness of an elderly more accurately.

## Data Availability Statement

The original contributions presented in the study are included in the article/supplementary material, further inquiries can be directed to the corresponding author/s.

## Ethics Statement

The studies involving human participants were reviewed and approved by Research Academy of Grand Health, Ningbo University (protocol code RAGH20190825). The patients/participants provided their written informed consent to participate in this study.

## Author Contributions

LX, QM, ZG, and YG worked for the conception and experiment design. LX, KD, and TY were involved in the data processing and manuscript writing. AW, JF, and YG helped in manuscript revision and final approval of the manuscript. All authors contributed to the article and agreed to the submitted version of the manuscript.

## Funding

This research was funded by the Natural Science Foundation of Zhejiang Province (LQ21H060003), Key Project of the National Social Science Foundation of China (19ZDA352), NSFC-RSE Joint Project (81911530253), Key R&D Program of Zhejiang Province China (2021C03130), Public Welfare Science and Technology Project of Ningbo, China (2021S133), Zhejiang Province Science Fund for Distinguished Young Scholars (R22A021199), and K. C. Wong Magna Fund in Ningbo University. LX is being sponsored by the China Scholarship Council (CSC).

## Conflict of Interest

The authors declare that the research was conducted in the absence of any commercial or financial relationships that could be construed as a potential conflict of interest.

## Publisher's Note

All claims expressed in this article are solely those of the authors and do not necessarily represent those of their affiliated organizations, or those of the publisher, the editors and the reviewers. Any product that may be evaluated in this article, or claim that may be made by its manufacturer, is not guaranteed or endorsed by the publisher.
